# Transcatheter interventions in critically ill neonates and infants with aortic coarctation

**DOI:** 10.4103/0974-2069.58312

**Published:** 2009

**Authors:** P Syamasundar Rao

Surgical correction of aortic coarctation (AC) was concurrently introduced by Crafoord and Nylin,[1] Gross and Hufnagel[2] in the early 1940s. Since that time surgical therapy has become the preferred treatment option for AC. Nearly three decades ago, Gruntzig’s technique of balloon angioplasty[3] was adopted by Sos,[4] Singer,[5] Sperling[6] and colleagues for enlargement of coarcted aortic segments in a postmortem specimen, postsurgical recoarctation, and native coarctation, respectively. Subsequently, other cardiologists used this technique to treat native AC.

Despite an initial report of poor results,[7] subsequent experience with balloon angioplasty appears encouraging and was reviewed elsewhere.[8‐12] The editorial critique on the results of the first successful case series of balloon angioplasty for native AC read[13]: “Now that we can dilate, should we?”[14] This was followed by reports of formation of aneurysms following balloon angioplasty of AC;[15,16] which in retrospect, may have been related to the use of very large balloons. The foregoing events cast skepticism on the usefulness of balloon angioplasty in the management of AC among pediatric cardiology and the cardiovascular surgical community. Shortly thereafter, the results of Valvuloplasty and Angioplasty of Congenital Anomalies (VACA) registry for native ACs[17] and post-surgical aortic recoarcatations[18] were published. Reduction in peak-to-peak systolic pressure gradient following balloon angioplasty for native coarctation was from 48 ± 19 mmHg to 12 ± 11 mmHg while that for post-surgical recoarctation was from 42 ± 20 mmHg to 9 ± 3 mmHg and these results were not statistically different (*P* > 0.05). The complication rates (9.9% vs. 8.5%) and mortality rates (0.7% vs. 2.5%) were also not different (*P* = 0.05 to 0.1).

The conclusion for native ACs was “the question remains not can it be done, but should it be done?,” whereas the conclusion for post-surgical aortic recoarctation was “... balloon angioplasty for relief of residual or recurrent aortic coarctation offers an acceptable alternative to repeat surgical repair” despite the fact that results were similar or even better for native AC. I questioned this interpretation[19] stating that “this is not logical and that objectivity of scientific interpretation should be maintained.” Subsequently, additional arguments advocating balloon angioplasty as a first-line therapeutic option in the management of native AC were presented.[20,21]

While treatment of native AC by balloon angioplasty was initially controversial,[14,17,19‐24] it gradually gained acceptance in the management of children with native coarctation. However, it remained controversial in the neonates and young infants.[22,25‐27]

In this issue of the Journal, Francis *et al*.[28] present data on 10 infants (mean age = 2.9 weeks) who underwent transcatheter intervention (balloon angioplasty in five and stents in five) for management of severe AC with left ventricular dysfunction. Peak systolic pressure gradient across the coarctation was reduced from 51 ± 12 mmHg to 8.7±6.7 mmHg. More importantly, the left ventricular dysfunction abated and patients were discharged home 6.5±3.4 days after procedure. However, all patients developed restenosis, requiring elective surgical repair or repeat balloon dilatation. The authors conclude that balloon dilatation with or without stent is an effective, though temporary, palliation for sick infants with severe AC. This is a well-written paper addressing the difficult issue of AC in neonates and young infants. However, the paper includes some issues worthy of further exploration: Authors have not clearly stated the reasons for choosing balloon angioplasty over stent. While they had seven patients with discrete narrowing and three had arch hypoplasia, five had stents implanted, meaning that they implanted stents in some patients with discrete coarctation. Although Lababidi *et al*.[13] were amongst the first to report balloon dilatation of AC, Sperling,[6] Lock,[7] Finley,[29] Brodsky,[30] Cooper[31] and Suarez de Lezo[32] had reported successful dilatation of AC earlier. They misquoted our papers[25,33] in that we preferred surgery over balloon angioplasty; indeed, our preference and recommendations were to use balloon angioplasty as first-line therapeutic option.[25,33] Finally, the authors[28] did not acknowledge prior reports in early 1990s in which Salauddin[34] and Rao[35] from our group reported use of balloon angioplasty for exactly the same reason Francis *et al*.[28] used transcatheter intervention. Despite these limitations, the authors’ data add value and supplement the previously reported experience with transcatheter intervention for AC. Furthermore, this paper provides an opportunity to discuss several issues germane to the management of coarctation of aorta in neonates and young infants.

## INDICATIONS

Indications for balloon angioplasty are similar to those used for surgical intervention: Significant hypertension and/or congestive heart failure. While the controversy over routine use of balloon angioplasty in treatment of neonatal and infant coarctations continues, it may certainly be utilized in special circumstances,[21] namely, neonates with shock-like picture and severe cardio-respiratory decompensation;[28,35] severe myocardial dysfunction, secondary to “hypertensive cardiomyopathy” (due to coarctation),[34] prior cerebral hemorrhage,[21] and liver dysfunction associated with biliary atresia.[21] We found balloon angioplasty to be useful in these situations and recommend it as first-line therapeutic option.

## SITE OF BALLOON CATHETER ENTRY

Potential for arterial damage exists, especially in neonates and young children. Therefore, use of umbilical artery approach in neonates[35‐38] and anterograde approach transvenously[37‐39] via a transposed aorta or through the ventricular septal defect, when feasible, should be undertaken. When femoral artery is catheter entry site, low profile balloons (for example Tyshak II, Minighost, MiniTyshak balloon catheters [Braun] and others) that can be introduced through 3 or 4 French sheaths should be utilized.

## BALLOON SIZE

The diameter of the balloon selected for angioplasty should be carefully chosen. Small balloons do not produce adequate relief of obstruction and large balloons may produce aortic rupture or aneurysm formation. Initial balloon dilatation is performed with a balloon whose diameter is an average of aortic isthmus or transverse aortic arch and descending aorta at the level of diaphragm. If there is no adequate relief of obstruction (i.e. gradient <20 mmHg) and angiographic improvement, repeat dilatation at the same sitting with a balloon as large as the diameter of the descending aorta at the level of diaphragm should be undertaken.[40] It is extremely rare that the balloon size should ever need to exceed the descending aortic size.

## POST-DILATATION CATHETER MANIPULATION

Tips of guide wires or catheters should not be manipulated over the freshly dilated coarctation segment to avoid aortic perforation.[29] A guide wire should always be left in place across the coarctation segment, and all angiographic and balloon-dilatation catheters should be exchanged over a guide wire.

## ARTERIAL INSUFFICIENCY

Evaluation of arterial insufficiency and limb growth retardation[41] in a group of children who underwent transfemoral artery interventions suggested compromised superficial femoral artery circulation without evidence of limb growth retardation during follow-up of one to eight years (mean, 3.5 years). Evaluation of arterial insufficiency by Doppler,[42] by measurement of ankle to brachial blood pressure index (ABI)[41] and/or by angiography,[41] should be incorporated into evaluation protocol during the follow-up for the primary cardiac condition.

## AORTIC STENTS

Stenotic vascular lesions can be dilated by balloon angioplasty. However, the elastic recoil of the vessel wall may return the vessel lumen to the pre-dilatation diameter following removal of the balloon catheter. Such recoil and vascular dissection, if any, following balloon dilatation can be circumvented with implantation of endovascular stents. Additional principles of stent management, historical aspects of stent development, types of stents, technique of stent deployment and results of stent therapy will not be reviewed here because of limits of space. The interested reader is referred elsewhere[43,44] for further discussion of these issues.

Because of growth issues and the need for large sheaths for stent implantation, most cardiologists limit stent usage to adolescents and adults, although a few have advocated their use in younger children.[45‐48] Even when large stents (for example, Palmaz P-8 series and Genesis XD) are used, they may not be dilatable to adult size aorta. Use of many of these stents may result in subjecting the patient to a more complicated surgical repair at a later date.[49] The small stents used by Francis *et al*.[28] indeed produced need for surgical intervention. Therefore, alternative solutions such as use of biodegradable stents[50,51] or growth stents[52] should be considered. Biodegradable stents keep the coarcted aortic segment open for a three to six month period, after which the stents dissolve. By then, the ratio of the normal aortic tissue to abnormal tissue may be in favor of the infant, thus preventing recurrence of significant narrowing. However, this hypothesis should be tested in appropriate animal models and stent delivery systems miniaturized so that they can be used in neonates and young infants. Similarly, growth stents may allow re-dilatation at a later date; although the limited experience is encouraging;[52] larger clinical trials are needed to confirm the utility of this concept.[53]

## CONCLUSIONS

In summary, the paper by Francis and associates which describes the utility of transcatheter intervention in neonates and young infants with critical coarctation of the aorta and left ventricular dysfunction, re-emphasizes prior reports advocating such approaches. Selection of site of entry for the procedure to minimize arterial damage, use of appropriate size balloon for angioplasty, avoiding manipulation of tips of catheters/guide wires across the freshly dilated coarctation segment, and incorporation of some method of evaluation of femoral artery sufficiency at the time of follow-up of the primary cardiac defect are germane to the success of the procedure. Avoiding use of stents in neonates and small infants and/or developing alternative strategies such as biodegradable stents or growth stents is recommended.
